# Electrosynthesis of >20 g/L H_2_O_2_ from Air

**DOI:** 10.1021/acsestengg.1c00366

**Published:** 2021-12-14

**Authors:** Huihui Li, Estefanny Quispe-Cardenas, Shasha Yang, Lifeng Yin, Yang Yang

**Affiliations:** † State Key Laboratory of Water Environment Simulation, School of Environment, Beijing Normal University, Beijing 100875, China; ‡ Department of Civil and Environmental Engineering, Clarkson University, Potsdam, New York 13699, United States

**Keywords:** gas diffusion electrode, hydrogen peroxide, electrosynthesis, cyanobacteria, microcystin

## Abstract

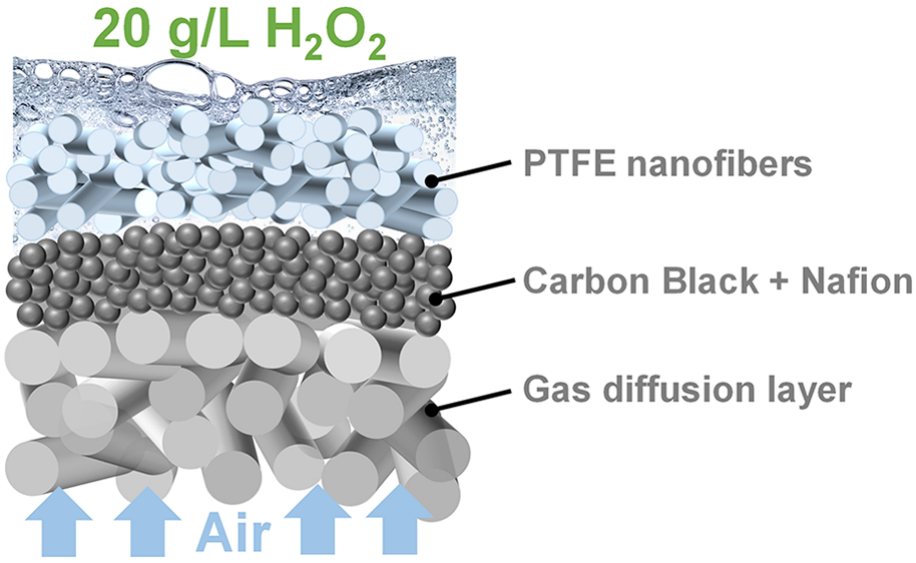

Hydrogen peroxide (HP) production via electrochemical oxygen reduction reaction (ORR-HP) is a critical reaction for energy storage and environmental remediation. The onsite production of high-concentration H_2_O_2_ using gas diffusion electrodes (GDEs) fed by air is especially attractive. However, many studies indicate that the air–GDE combination could not produce concentrated H_2_O_2_, as the [H_2_O_2_] leveled off or even decreased with the increasing reaction time. This study proves that the limiting factors are not the oxygen concentration in the air but the anodic and cathodic depletion of the as-formed H_2_O_2_. We proved that the anodic depletion could be excluded by adopting a divided electrolytic cell. Furthermore, we demonstrated that applying poly(tetrafluoroethylene) (PTFE) as an overcoating rather than a catalyst binder could effectively mitigate the cathodic decomposition pathways. Beyond that, we further developed a composite electrospun PTFE (E-PTFE)/carbon black (CB)/GDE electrode featuring the electrospun PTFE (E-PTFE) nanofibrous overcoating. The E-PTFE coating provides abundant triphase active sites and excludes the cathodic depletion reaction, enabling the production of >20 g/L H_2_O_2_ at a current efficiency of 86.6%. Finally, we demonstrated the efficacy of the ORR-HP device in lake water remediation. Cyanobacteria and microcystin-LR were readily removed along with the onsite production of H_2_O_2_.

## Introduction

Hydrogen peroxide (H_2_O_2_, HP) is an important chemical in various industries, including organic synthesis, pulp or textile bleaching, wastewater treatment, energy conversion, and other applications.^1^ The annual global production of H_2_O_2_ is about 4.5 million tons in 2020 and is projected to reach 5.7 million metric tons by the year 2027.^2^ It is mainly produced by an anthraquinone process,^3^ which consists of sequential hydrogenation, oxidation, and distillation to produce concentrated H_2_O_2_.^4^ This method demands significant amounts of energy and generates considerable volumes of solvent wastes.^5^ Besides, the transport and storage of concentrated H_2_O_2_ for water treatment could incur high costs and safety concerns.^6^ It is also undesired considering that only diluted concentration (usually <0.1 wt %) is needed.^7^

The onsite production of H_2_O_2_ by the two-electron oxygen reduction reaction (ORR-HP) (eq 1) emerged as an alternative approach.^8^ The electrochemical reactor for ORR-HP must be selective to avoid the competitive four-electron reduction reaction to produce water (eq 2).
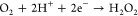
1
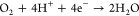
2Many of the studies in this area have been focused on the development of novel catalysts. Noble metals and alloys (*e.g*., Pd, Pd–Au, and Pd–Hg) show good reactivity toward ORR-HP at current efficiencies ranging from 80 to 96%.^9,10^ Carbonaceous catalysts were also extensively investigated as more affordable alternatives.^11,12^ Delicate active site fabrication approaches have been explored. Introducing oxygen-containing functional groups by oxidative pretreatment improved the ORR-HP activities of carbonaceous catalysts (carbon black (CB), carbon nanotube, graphene, *etc*.).^12−14^ More recently, the construction of atomically dispersed nitrogen or metal cation–nitrogen centers (M–N*_x_*) on carbon materials is gaining increasing momentum as well.^15,16^

Fundamental studies on novel catalyst development are imperative for gaining a deeper understanding of reaction mechanisms and pushing the limit of the current efficiency of ORR-HP toward the ultimate goal of 100%. However, from a practical engineering perspective, the moderate reactivity (*e.g*., 80–90% current efficiency) of carbon black (CB) is already acceptable for onsite H_2_O_2_ production, considering its low cost and accessibility.

Even if CB is adopted to reduce the system cost, the remaining challenge is producing concentrated H_2_O_2_. An ORR-HP system can be driven by purging oxygen or air into the water.^15−17^ However, neither the storage of pure oxygen nor the forced air convection (*i.e*., by compressors) is desired in the field due to safety and energy consumption concerns. Alternatively, the gas diffusion electrodes (GDEs) were developed. The GDE comprises an air-facing gas diffusion layer and a water-facing CB layer. Gas can spontaneously diffuse to the catalyst layer to drive the ORR-HP reaction. However, most CB/GDE/air systems showed that the increase of [H_2_O_2_] became sluggish and leveled off at ceiling concentrations (<1 g/L; see summary in Table S1) despite the extension of reaction time.^18−20^

So far, there is no clear explanation of what factors prohibit the production of concentrated H_2_O_2_. To answer the pending question; first, a theoretical validation is needed to prove that oxygen in the air by diffusion is sufficient to sustain the production of concentrated H_2_O_2_. Second, it is critical to investigate whether the parallel anodic or cathodic H_2_O_2_ reactions jeopardize the build-up of [H_2_O_2_]. Lastly, the corresponding solutions could be developed by optimizing the configurations of cells and GDEs.

Following the above research approaches, we optimized the cell configuration and developed a novel GDE cathode featuring the electrospun poly(tetrafluoroethylene) (E-PTFE) coating on top of, rather than blending with, the CB layer. The electrospun PTFE nanofibers provide abundant gas/water/catalyst triphase active sites and prevent the decomposition of the as-formed H_2_O_2_ and realize the production of >20 g/L H_2_O_2_.

## Experimental Section

### Chemicals and Materials

Poly(tetrafluoroethylene) (PTFE, 60 wt %), cation-exchange membrane (Nafion 117, 183 μm), Nafion dispersion (D520, 5 wt %), carbon black (XC72) powder, and graphite powder were purchased from Fuel Cell Store. Poly(ethylene oxide) (PEO, *M*_w_ = 100 000 g/mol) and hydrogen peroxide (30 wt %) were obtained from Sigma-Aldrich. Potassium titanium oxide oxalate dihydrate (K_2_[TiO(C_2_O_4_)_2_·2H_2_O], concentrated nitric acid (HNO_3_), concentrated sulfuric acid (H_2_SO_4_), and sodium perchlorate (NaClO_4_) were obtained from Fisher Scientific. Besides, 2-propanol was purchased from J. T. Baker. The carbon paper (Sigracet 39 BB, 10 cm × 10 cm) was purchased from Fuel Cell Store. The oxygenated carbon black (O-CB) was prepared by the acid-reflux method.^21^ Briefly, 300 mg carbon black (XC72) was refluxed with 300 mL of 12 M nitric acid at 85 °C for 3 h. The iridium oxide (IrO_2_; 6 cm^2^) dimensionally stable anode was purchased from Entrustech, China. The cyanobacteria strain, *Microcystis aeruginosa* (UTEX LB 3037), was obtained from the University of Texas at Austin. The microcystin-LR (MC-LR) (CAS 101043-37-2) was provided by Millipore Sigma.

### Preparation of CB/GDE and PTFE-CB/GDE

Unless noted otherwise, Nafion was used as the binder. The catalyst ink was prepared by sonicating CB powder (12.5 mg) with 5 wt % Nafion (0.54 mL) and 2-propanol (0.46 mL) for 30 min. Then, the ink was sprayed onto the carbon paper using an airbrush (APR150 Amazon). The coated carbon paper (CB/GDE) was vacuum-dried in a vacuum oven at 60 °C and then sintered at 350 °C for 40 min. The loading amount of CB can be controlled by adjusting the volume of ink applied. For comparison, PTFE was used as a binder to prepare PTFE-CB/GDE following the reported method with modification:^6^ 12.5 mg of CB powder was sonicated with 1 mL of 0.4% PTFE solution for 1 h. The [CB]/[PTFE] mass ratio is 3:1. The ink was then spray-coated on carbon paper. The resultant sample “PTFE-CB/GDE” was dried and sintered as mentioned above.

### Preparation of PTFE/CB/GDE and E-PTFE/CB/GDE

Advanced from the conventional PTFE-CB/GDE, this study proposed a new configuration in which PTFE should be coated on, not blended with, the CB layer. Specifically, PTFE (20 wt %) solution was spray-coated on CB/GDE. The loadings were controlled by spraying duration. After that, the PTFE/CB/GDE was vacuum-dried and sintered at 350 °C for 40 min. As a more advanced form, PTFE nanofibers were electrospun on CB/GDE using an electrospinning and electrospraying unit (MSK-NFES-3, MTI). The precursor solution was prepared by sonicating PEO (0.55 g) and PTFE (4.64 mL, 60 wt %) in DI water (2.45 mL) for 30 min.^22^ The precursor solution was transferred to a syringe (20 mL) placed in a syringe pump. The CB/GDE was fixed on an aluminum plate facing the syringe needle. A voltage of 20 kV was applied between the needle tip and plate at a distance of 15 cm. The pump flow rate was set as 0.5 mL/h. The electrospun PTFE (E-PTFE) fibers were deposited on the CB/GDE target. The electrospinning synthesis was operated for 30, 60, and 90 min to obtain different E-PTFE loadings. Finally, the E-PTFE/CB/GDEs were dried and sintered the same as above.

### Electrosynthesis of H_2_O_2_

The electrosynthesis of H_2_O_2_ was performed at room temperature (20 ± 2 °C) in an undivided or divided cell. For an undivided cell, 30 mL of 100 mM NaClO_4_ was used as the electrolyte. The distance between an IrO_2_ anode and a GDE cathode (12.6 cm^2^) is 4 cm. As for tests in a divided cell, a proton-exchange membrane was placed between anode and cathode distanced by 4 cm. The anolyte is 100 mM H_2_SO_4_ (30 mL). The catholyte is 30 mL of 100 mM NaClO_4_ or lake water.

### Analytical Methods

The H_2_O_2_ concentration was measured by the potassium titanium oxalate spectrophotometric method:^1^ 500 μL of diluted sample was mixed with 500 μL of K_2_[TiO(C_2_O_4_)_2_·2H_2_O (0.14 M) and 500 μL of H_2_SO_4_ (3 M). Absorbance at 400 nm was used to quantify [H_2_O_2_]. Electrochemical impedance spectroscopy (EIS) measurement was performed in the electrolyte containing 120 mM K_3_Fe(CN)_6_, 120 mM K_3_Fe(CN)_6_, and 330 mM KCl over the frequency range of 0.1 Hz to 100 kHz with a 10 mV sine wave.^23^ In the study of lake water treatment, chlorophyll-*a* samples were filtered, extracted, and measured by a fluorometer (TD-700, Turner Designs) according to the Welschmeyer method.^24^ The MC-LR was measured by a ultra-high-performance liquid chromatography (UPLC) system coupled with a triple-stage quadrupole mass spectrometer (Thermo Scientific, Vanquish-TSQ ALTIS) equipped with a Phenomenex Luna Omega PS C18 column (1.6 μm, 100 × 2.1 mm^2^).

## Results and Discussion

### Using Air as an Oxygen Source

As a theoretical groundwork for this study, the first step is to prove that oxygen in the air (*P*_O_2__ = 0.21 atm) is sufficient to produce concentrated H_2_O_2_. A back-of-the-envelope calculation was performed assuming that the current efficiency of ORR-HP is 100% and the cross-diffusion-layer mass transfer is the only rate-limiting step. In theory, the flux of electrons (*i.e*., current density) should be proportional to the oxygen flux diffused across the GDE. Thus, the limiting current (*I*_limt_) can be calculated as follows^25^
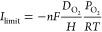
3where *n* = 2 is the number of electrons in the ORR-HP reaction, *F* is the Faraday constant (96 485 C/mol), *D*_O_2__ (1.90 × 10^–5^ m^2^/s) is the oxygen diffusion coefficient in the gas diffusion layer,^26^*H* is the thickness of the gas diffusion layer (measured as 3.15 × 10^–4^ m), *P*_O_2__ (21 278 Pa) is the oxygen partial pressure in the air, *R* is the universal gas constant (8.314 J/(mol·K)), and *T* (298 K) is the gas temperature.

The limiting current density is calculated as 10 A/cm^2^. As an extreme case, 30 mL of commercial 35 wt % H_2_O_2_ can be readily synthesized in 11 min using a 10 cm^2^ GDE at the limiting current density (Text S1). The above calculation suggests that air by diffusion can sustain the synthesis of concentrated H_2_O_2_. Therefore, neither pure oxygen nor forced aeration should be required.

### Single *vs* Divided Cell

Knowing that ORR-HP production is not limited by oxygen concentration in air, the next step is to evaluate the cell configuration. Previously, ORR-HP reactions have been performed in either single or divided cells.^19,27,28^ Controversial reports showed that H_2_O_2_ could be decomposed or produced by anodic reactions.^29,30^ We believe it is pivotal to unbiasedly compare the ORR-HP performances of single and divided cells under a uniform test condition.

Electrosynthesis of H_2_O_2_ was performed in a single cell (30 mL) equipped with the CB/GDE cathode (CB loading 0.5 mg/cm^2^). Preliminary studies found that [H_2_O_2_] linearly increased with the electrolysis duration within the initial 10 min. Therefore, the H_2_O_2_ evolution rates (d[H_2_O_2_]/d*t*) at different current densities were used to calculate the current efficiency (eq 4).
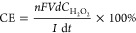
4where *n* = 2 is the number of electrons transferred for oxygen reduction to H_2_O_2_, *F* is the Faraday constant (96 486 C/mol), *C*_H_2_O_2__ is the concentration of H_2_O_2_ (mol/L), *V* is the volume of catholyte (L), *I* is the current (A), and *t* is the time (s).

**Figure 1:**
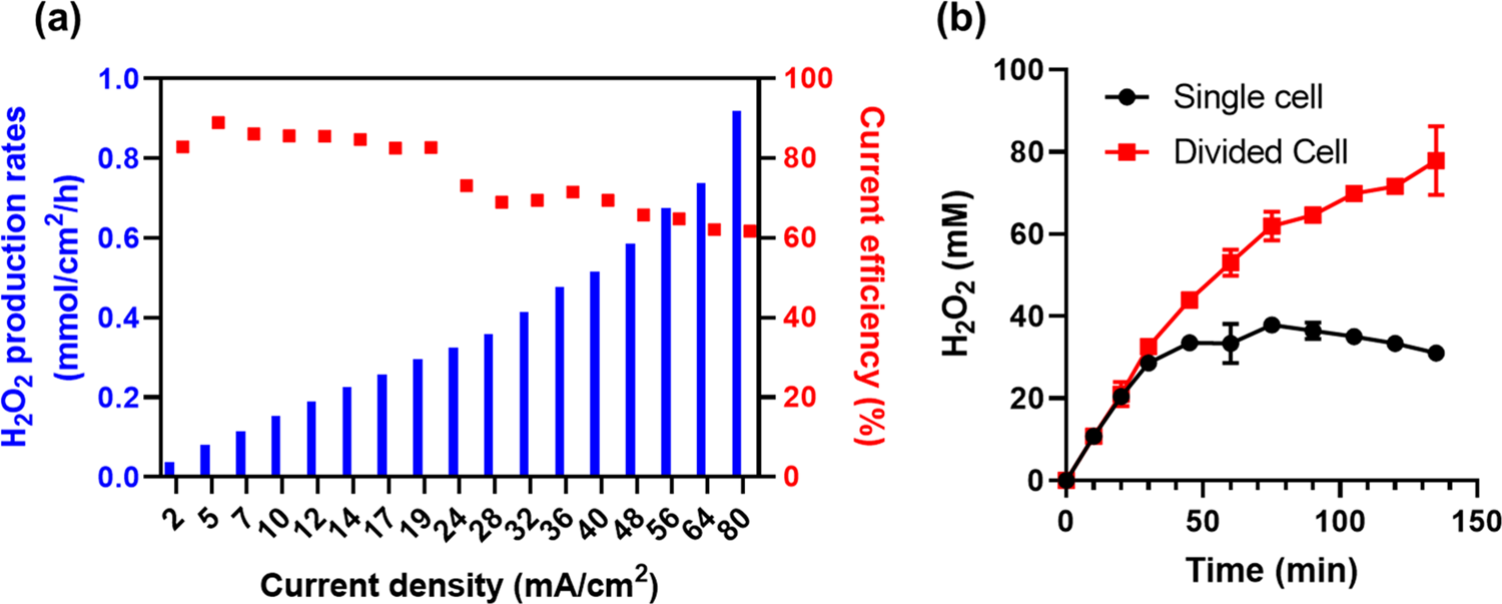
(a) H_2_O_2_ evolution rates and current efficiencies at different current densities in a single cell. The reaction was performed in 30 mL of 100 mM NaClO_4_ using a 12.6 cm^2^ CB/GDE. (b) Comparison of ORR-HP performances between single and divided cells. The ratio of [cathode area: 12.6 cm^2^]/[electrolyte volume: 30 mL] in the cathodic chamber of the divided cell is the same as that in the single cell. Electrolysis was performed at 12 mA/cm^2^.

Current densities at 2–80 mA/cm^2^ far below the theoretical limiting current (10 A/cm^2^) were tested. The CB catalyst already exhibited more than 80% current efficiency at 2–19 mA/cm^2^ (Figure 1a), comparable to the state-of-art catalysts (rare earth nanorods, nitrogen-doped carbon) prepared by more delicate procedures.^31,32^ An increase in current density results in the elevation of the H_2_O_2_ evolution rate. However, current efficiency decreases due to the competitive four-electron ORR to produce H_2_O (eq 2).

A current density of 12 mA/cm^2^ balancing the H_2_O_2_ evolution rate and current efficiency was used in the following studies. Theoretically, if H_2_O_2_ can be continuously produced in a batch reactor without depletion, any desired high concentrations could be achieved. However, long-term electrolysis performed in the single cell at 12 mA/cm^2^ shows that [H_2_O_2_] leveled off after 60 min at 37 mM (Figure 1b). For comparison, the ORR-HP reaction was conducted in a divided cell using a proton-exchange membrane (Nafion 117) to separate a cell into anodic and cathodic chambers. The [cathode area: 12.6 cm^2^]/[electrolyte volume: 30 mL] ratio of the cathodic chamber is the same as that of the single cell to enable unbiased comparison.

The change in cell configuration did not impact the initial H_2_O_2_ production rate (Figure 1b). However, the divided cell was able to build up higher [H_2_O_2_] than a single cell with the extended reaction time, indicating that the anodic oxidation of H_2_O_2_ (O_2_ + 2H^+^ + 2e^–^ → H_2_O_2_; *E*^0^ = 0.695 V) in a single cell leads to the loss of the as-formed H_2_O_2_.^29^ Although the divided cell outperformed single cell, the further increase of [H_2_O_2_] became sluggish after 100 min, indicating the concurrence of cathodic H_2_O_2_ depletion. Benzoic acid (1 mM) was spiked in the catholyte as a radical probe.^33,34^ No degradation of BA was observed throughout 135 min of electrolysis. Therefore, the conversion of H_2_O_2_ to ^•^OH was excluded, leaving the most plausible H_2_O_2_ depletion mechanism as the cathodic reduction of H_2_O_2_ by CB (H_2_O_2_ + 2H^+^ + 2e^–^ → 2H_2_O; *E*^0^ = 1.76 V).^35^

### PTFE/CB/GDE *vs* PTFE-CB/GDE

**Figure 2:**
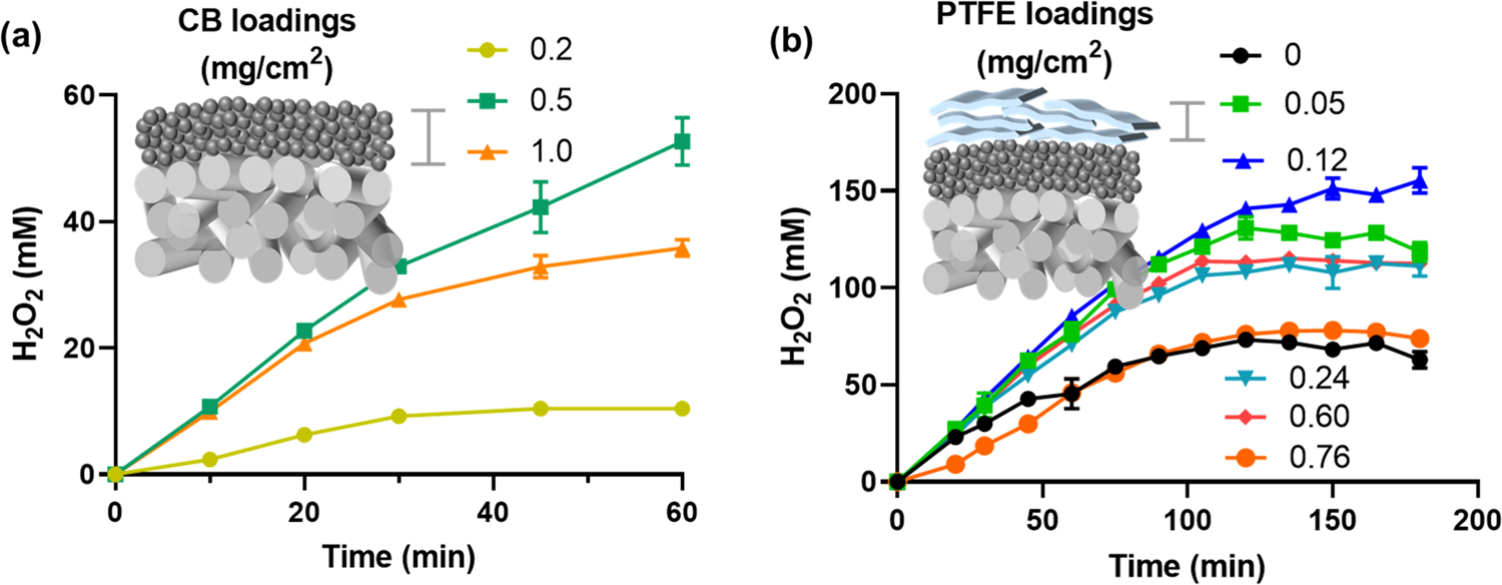
Effect of the (a) CB loading and (b) PTFE loading on the production and accumulation of H_2_O_2_. PTFE was loaded onto the CB layer by spray coating. Current density = 12 mA/cm^2^.

Given the above, the rationale design of GDE is imperative to mitigate the cathodic decomposition of the as-formed H_2_O_2_. Therefore, we first worked on the optimization of CB loadings. It was found that increasing the CB loading from 0.2 to 0.5 mg/cm^2^ facilitates H_2_O_2_ production (Figure 2a). Up to 55 mM H_2_O_2_ was produced after 60 min reaction by a CB/GDE with CB loading of 0.5 mg/cm^2^. Further increase in the CB loading to 1 mg/cm^2^ leads to a similar initial production rate within the initial 10–20 min, but the [H_2_O_2_] levels off at 36.8 mM after 40 min. These results suggest a trade-off between catalyst loading and H_2_O_2_ production efficiency: higher CB loadings provide more active sites for ORR-HP reactions, but excessive active sites could also accelerate the cathodic reduction of H_2_O_2_.

We suspected that the ORR-HP reactions occurred at the CB/GDE interface. The as-formed H_2_O_2_ is subsequently transported through the CB/electrolyte interface, where H_2_O_2_ decomposition could also occur. We also hypothesized that a hydrophobic CB/electrolyte interface could minimize the contact between the as-formed H_2_O_2_ and CB and consequently prevent decomposition.

To validate this assumption, the CB layer was spray-coated with different amounts of PTFE to increase the hydrophobicity. The composite cathode is denoted as PTFE/CB/GDE. The peak [H_2_O_2_] was successfully increased from 70 mM without PTFE modification to 150 mM with 0.12 mg/cm^2^ PTFE loading (Figure 2b). However, further increasing the PTFE loading blocked the CB layer from the electrolyte, evidenced by the dramatic increase of cell voltage (Figure S1).

It is important to emphasize that the PTFE/CB/GDE proposed in this study is fundamentally different from the previous GDE using PTFE as the bulk CB catalyst binder (denoted as PTFE-CB/GDE).^6,7,36^ For the conventional PTFE-CB/GDE, PTFE was blended with CB to provide bulk hydrophobicity. As for the PTFE/CB/GDE cathode, PTFE was only applied on the surface, while the CB layer beneath used the hydrophilic Nafion polymer to promote proton transfer.^37^

Figure S2 demonstrates that PTFE/CB/GDE outperforms PTFE-CB/GDE in terms of the initial production rate and maximum concentration (150 *vs* 50 mM). The superior performance of PTFE/CB/GDE could be assigned to the synergy of the proton-conductive CB layer, which effectively produces H_2_O_2_, and the hydrophobic surface, which minimizes contact between the as-formed H_2_O_2_ and the CB layer to avoid decomposition. In contrast, the proton transfer of PTFE-CB/GDE is less efficient, leading to inferior ORR-HP performance (more evidence will be presented in Figure 6).

Some research demonstrated that introducing oxygen-containing functional groups to CB could promote H_2_O_2_ production.^38^ Therefore, the CB layer in the GDE/CB/PTFE cathode was replaced with the equivalent mass loading of oxygenated CB (O-CB) prepared by the HNO_3_ reflux method.^21^ However, we found that O-CB neither promoted the H_2_O_2_ production nor reduced the cell voltage (Figure S3).

### EP-PTFE/CB/GDE Advanced from PTFE/CB/GDE

The hydrophobic spray-coated PTFE elevates the peak [H_2_O_2_] from 55 to 155 mM. However, flooding of the GDE and the flake-off of PTFE coatings were also noticed, leading to the retarded growth of [H_2_O_2_] after 150 min (Figure 2b). The results suggest that spray-coated PTFE is neither uniform nor mechanically durable. The long-term operation will expose the underneath CB layer to favor the cathodic decomposition of H_2_O_2_.

Therefore, an ideal PTFE coating should be hydrophobic, water-permeable (to access CB layer), and durable. Recently, the rationale design of the electrode/electrolyte/gas triphase interface has gained enormous research thrust.^39−41^ For example, the coating of a pervious hydrophobic polymer layer on top of a hydrophilic catalyst layer could produce abundant air/water/catalyst triphase interfaces, leading to a record-high CO_2_ reduction reactivity.^40^ Neither such strategy was adopted nor the polymer coating was developed for the ORR-HP process. We, therefore, believe that turning the PTFE coating to a permeable structure could further improve the performance of PTFE/CB/GDE.

In light of the above hypothesis, the electrospinning method was used to weave PTFE (E-PTFE) nanofibers on the CB catalyst layer to form a self-standing hydrophobic fabric with good mechanical strength and abundant voids between nanofibers to provide triphase interfaces.

**Figure 3:**
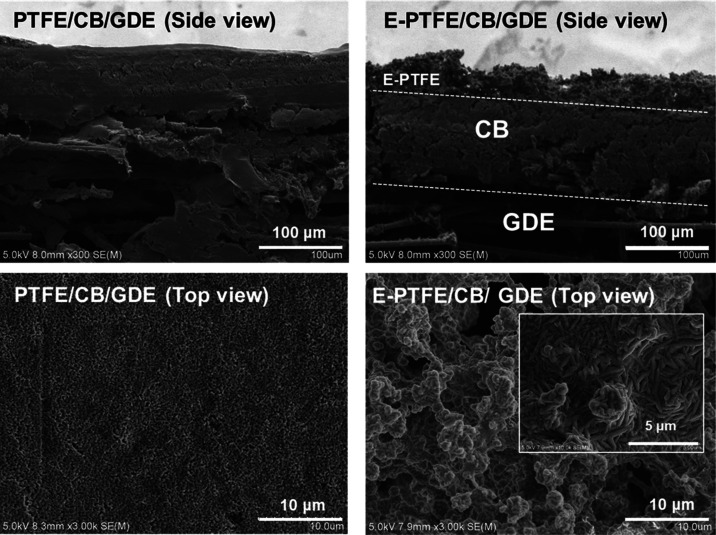
Top and side view of the composite GDE cathodes.

The morphology of E-PTFE/CB/GDE was examined by scanning electron microscopy (SEM; Hitachi X650, Japan) (Figure 3). The E-PTFE forms porous structures on the CB layer as expected. In contrast, the spray-coated PTFE forms a dense layer that cannot be distinguished from the CB layer.

**Figure 4:**
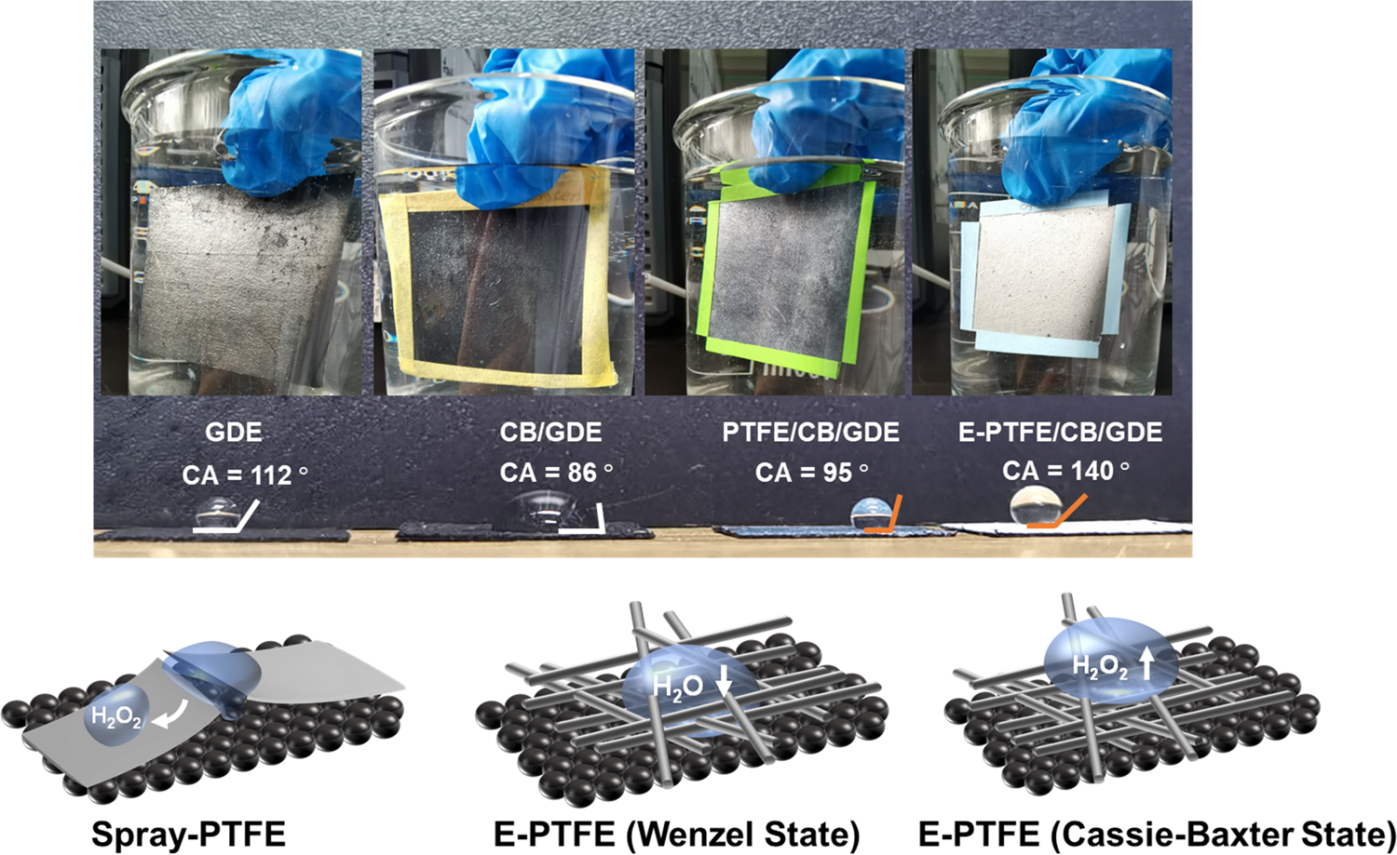
Photographic images of the submerged pristine and modified GDE cathodes and contact angles of cathodes. CB loading: 0.5 mg/cm^2^; PTFE loading: 0.12 mg/cm^2^; E-PTFE loading: 1 mg/cm^2^. Silvery reflection was observed on E-PTFE/CB/GDE due to the formation of a continuous air film. The lower panel demonstrates the possible ways of entry of water at the PTFE/electrolyte interface. For better demonstration, water was visualized as droplets. In reality, water should act as a bulk phase covering the cathode in the ORR-HP reactions.

Figure 4 compares the hydrophobicity of different GDEs. The spray-coated PTFE increases the water contact angle from 86 to 95°. The coating of E-PTFE further elevates the contact angle to 140°.

**Figure 5:**
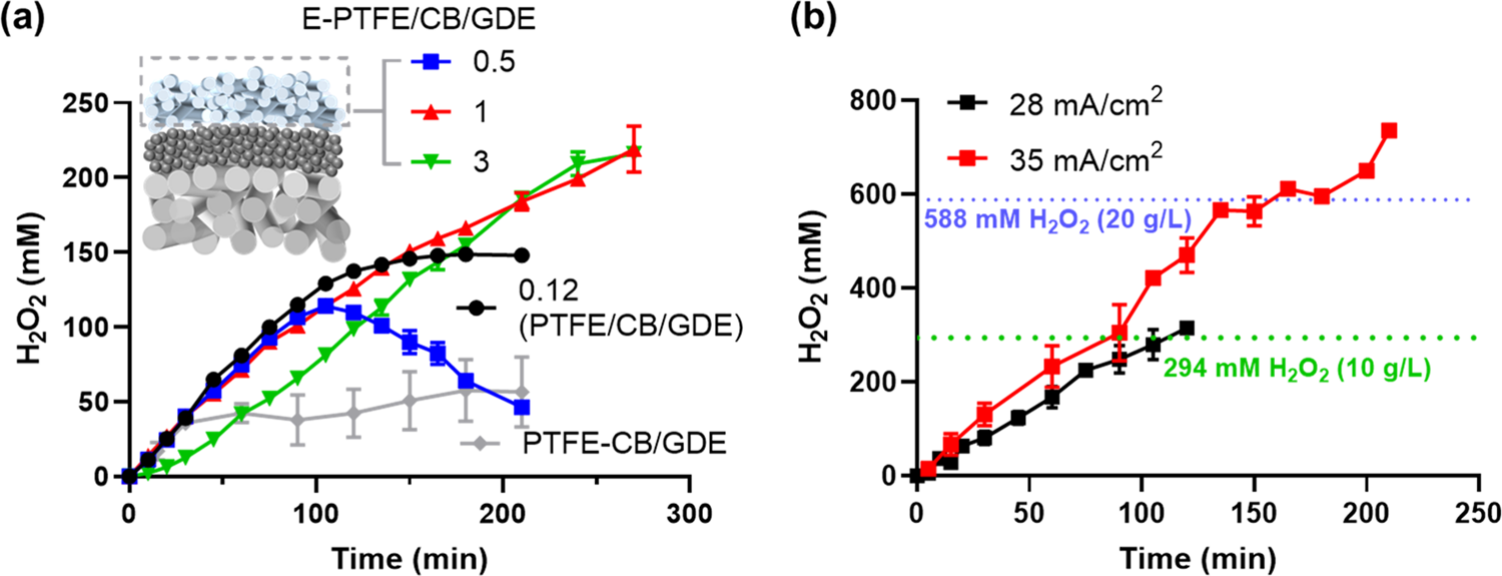
(a) Effect of the loadings of the electrospun PTFE on the H_2_O_2_ production and accumulation at 12 mA/cm^2^. Numbers indicate the loadings of PTFE (electrospun or spray-coated). The CB mass loading of all samples is 0.5 mg/cm^2^. (b) E-PTFE (1 mg/cm^2^)/CB/GDE operated at 28 and 35 mA/cm^2^ to produce concentrated H_2_O_2_. Catholyte: 0.1 M NaClO_4_. Anodolyte: 0.1 M H_2_SO_4_.

Figure 5a demonstrates the impacts of E-PTFE loadings on the ORR-HP reactions. A 0.5 mg/cm^2^ E-PTFE overcoating cannot prevent the flooding of GDE after 105 min of operation. The cathodic depletion pathway prevailed over H_2_O_2_ production, leading to the decrease of [H_2_O_2_]. We then increased the loading of E-PTFE by extending the electrospun duration. The cathode with E-PTFE loading of 1 mg/cm^2^ could produce 200 mM H_2_O_2_ with no sign of retardation. Further increasing the E-PTFE from 1 to 3 mg/cm^2^ did not impair HP production but raised the cell voltage from 4.7 to 5.7 V (Figure S4). Therefore, 1 mg/cm^2^ was identified as the optimum loading of E-PTFE. Most importantly, E-PTFE/CB/GDE showed superior performance than PTFE/CB/GDE and PTFE-CB/GDE, as indicated by the continuous build-up of [H_2_O_2_] with the extended reaction time.

The E-PTFE (1 mg/cm^2^)/CB/GDE cathode was operated at a higher current density to accelerate the H_2_O_2_ production. The divided cell operated at 35 mA/cm^2^ can readily produce 735 mM (25 g/L) H_2_O_2_ (Figure 5b). In another set of tests, 200 mM H_2_O_2_ was spiked in the electrolyte before electrosynthesis at various current densities (Figure S5). The continuous increase of [H_2_O_2_] beyond 200 mM implies that cathodic depletion of HP was successfully eliminated on E-PTFE/CB/GDE. The durability of E-PTFE/CB/GDE was challenged in six rounds of repetitive tests at 28 mA/cm^2^. No sign of activity loss nor structural damage was observed in these tests (Figure S6).

It is important to highlight that the E-PTFE/CB/GDE composite cathode is noble-metal-free and made by a facile process. Air was used as the only oxygen source, and no force gas convection was required. Table S1 summarizes the performance of representative GDE studies (since 2008). The H_2_O_2_ production rate (0.57 mmol/(cm^2^ h)) and current efficiency (86.8%) observed in this study are exceeding or commensurate with that of the systems using pure oxygen or delicately prepared catalysts (graphene, carbon nanotubes, nitrogen/oxygen-doped carbon, *etc*.)

The variation of cell voltages (Figure S4) during the ORR-HP process provides critical mechanistic insights. For the CB/GDE cathode, the cell voltage gradually increased from 4 to 5.1 V. In contrast, the PTFE-coated cathodes have high initial cell voltages. For instance, the initial cell voltage of E-PTFE/CB/GDE is 8 V and then gradually decreased to 4.8 V. Since all of the tests were performed in the same cell, the cell voltages are only determined by the internal resistance of cathodes. The initial high cell voltage of PTFE-coated cathodes could be attributed to an insulative air film above the PTFE coating. When current was applied, the air film was consumed, leading to decreased cell voltages.

Combining the information collected from SEM imaging and contact angle measurement, we speculate that for PTFE/CB/GDE, the CB catalysts access the bulk electrolyte through the cracks of PTFE coating (Figure 4). The produced H_2_O_2_ will then be expelled from the cathode by the hydrophobic PTFE coating. This hypothesis explains that PTFE/CB/GDE has a good capability to preserve H_2_O_2_ than the CB/GDE cathode.

The E-PTFE/CB/GDE has a larger contact angle than PTFE/CB/GDE, implying that factors in addition to the PTFE hydrophobic functional groups (*i.e*., fluorocarbon chain) contribute to the enhanced hydrophobicity. This phenomenon can be explained as the water landed on the PTFE-weaved fibers is supported by air pockets partially (Wenzel state) or completely (Cassie–Baxter state) (Figure 4).^42,43^ The E-PTFE/CB/GDE with higher ORR-HP performance has a lower steady-state cell voltage than PTFE/CB/GDE (4.8 *vs* 5.6 V in Figure S4). These results imply that the CB layer of E-PTFE/CB/GDE is more accessible to electrolytes than PTFE/CB/GDE. Thus, E-PTFE/CB/GDE has lower internal resistance. We hypothesize a dynamic equilibrium between the Wenzel and Cassie–Baxter states at the electrode/electrolyte interface. As a result, water and air trapped between fiber gaps closely contact the CB catalyst layer to produce H_2_O_2_ (the Wenzel state in Figure 4). The as-formed H_2_O_2_ is then expelled by the air pockets replenished by air diffused from the backside of the GDE (Cassie–Baxter state in Figure 4). Figure S7 shows that removing dissolved oxygen in the electrolyte by nitrogen purging did not impact the H_2_O_2_ production, supporting that oxygen originated from the air-facing side of GDE.

**Figure 6:**
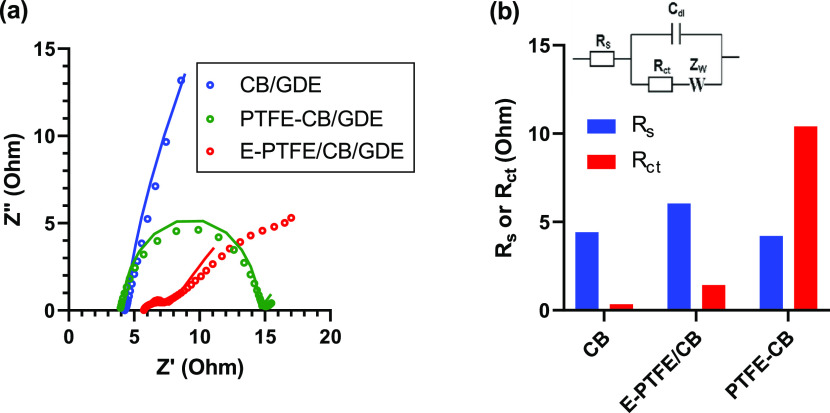
(a) Nyquist plots of different cathodes. Dots and lines represent experimental data and fitted results, respectively. (b) Comparison of fitted *R*_s_ and *R*_ct_ values of different GDEs.

We further perform EIS analysis to explain the superior performance of E-PTFE/CB/GDE compared with the conventional PTFE-CB/GDE and CB/GDE. The Nyquist plots of E-PTFE/CB/GDE, CB/GDE, and PTFE-CB/GDE (Figure 6a) were fitted by the Randles circuit, which gives the fitted values of solution resistance (*R*_s_) and charge-transfer resistance (*R*_ct_) shown in Figure 6b. E-PTFE/CB/GDE has higher *R*_s_ than CB/GDE and PTFE-CB/GDE, indicating the existence of continuous air film, which elevates the internal resistance of the cathode. On the other hand, E-PTFE/CB/GDE has a lower *R*_ct_ than PTFE-CB/GDE. The result implies that the former cathode has excellent ion (proton) transfer efficiency within the CB layer^44^ due to the use of the Nafion binder.

With the above finding, we conclude that E-PTFE/CB/GDE featuring a pervious hydrophobic surface and ionic conductive inner catalytic layer is the best-performing cathode for ORR-HP reactions to support the following environmental application.

### Environmental Application

**Figure 7:**
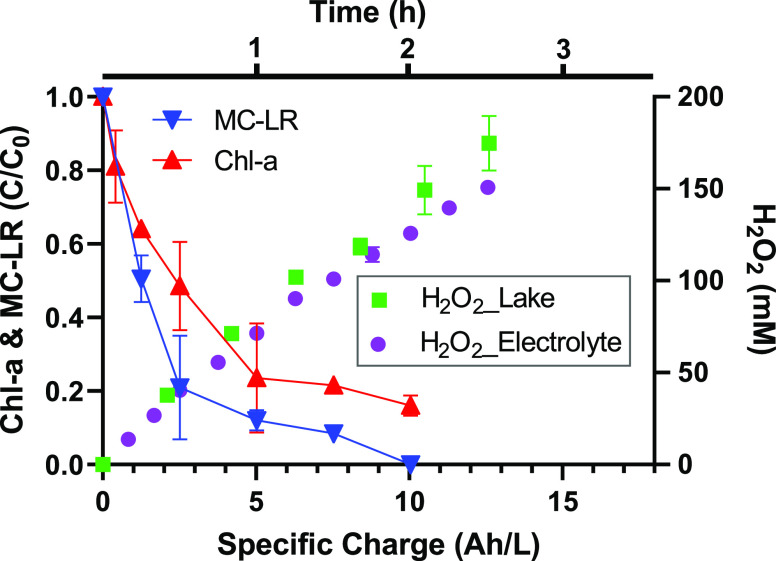
In situ H_2_O_2_ production to inactivate cyanobacteria and destroy microcystin-LR in lake water. The initial concentrations of cyanobacteria (characterized as chlorophyll-*a* concentration [Chl-*a*]) and microcystin-LR (MC-LR) are 100 and 1 μg/L, respectively. The divided cell was operated at 12 mA/cm^2^ and voltage of 15 V.

The best-performing E-PTFE (1 mg/cm^2^)/CB (0.5 mg/cm^2^)/GDE was used to produce H_2_O_2_ in lake water (Figure 7). Lake water (30 mL; pH 7.0, conductivity: 1068 μS/cm) was used as a catholyte. H_2_SO_4_ (0.1 M) was used as an anolyte. The linear increase of [H_2_O_2_] to 150 mM was observed after 2 h. The H_2_O_2_ production rate in lake water is commensurate with that in the synthetic electrolyte (100 mM NaClO_4_).

The energy consumption for H_2_O_2_ production in 100 mM NaClO_4_ electrolyte is 15 kWh/kg (Text S2), which is within the range of the reported laboratory results (6.0–22.1 kWh/kg).^45^ The production of H_2_O_2_ in lake water with lower conductivity (1068 μS/cm) leads to the higher energy consumption of 32 kWh/kg (Text S2). These results suggest that the high conductivity of the water is the key to lowering energy consumption by reducing the ohmic loss in the electrolyte. Therefore, the ORR-HP process could be more energy efficient for treating seawater, filtration concentrate, and leachate. For the treatment of the less conductive water, engineering solutions to reduce the ohmic loss include (1) the reduction of electrode spacing and (2) packing the cathodic chamber with ion-exchange resin as the solid electrolyte to facilitate ion conduction.^46^

Nevertheless, we demonstrated that the current ORR-HP system could be applied to mitigate harmful algal blooms. Cyanobacteria (100 μg/L as Chl-*a*) and microcystin-LR (1 μg/L) spiked in lake water were effectively removed by H_2_O_2_ produced within 1 h, corresponding to a specific charge of 5 Ah/L.

It is important to emphasize that this article mainly focuses on system optimization and electrode material development to produce concentrated H_2_O_2_. The 10 or 20 g/L benchmarks (Figure 5b) were set arbitrarily to demonstrate the superior performance of E-PTFE/CB/GDE cathode among peer studies (Table S1). In typical advanced oxidation processes, much lower [H_2_O_2_] (usually <1 g/L) is required.^7^ In practical application, a side stream of water can be diverted to the ORR-HP unit, and then the effluent could carry over high-concentration H_2_O_2_ to the mainstream. The activation of H_2_O_2_ to generate ^•^OH by photolysis and Fenton catalysts was extensively documented and thus not included in this single article.

## Conclusions

This study demonstrated step-wise approaches to identify and exclude H_2_O_2_ depletion pathways in the ORR-HP system. The anodic decomposition of H_2_O_2_ was excluded by adopting a divided cell configuration. More importantly, we found that PTFE overcoated on, rather than blended in, the CB catalyst could significantly promote the H_2_O_2_ production activity and exclude the cathodic H_2_O_2_ decomposition. Beyond this finding, PTFE overcoating was fabricated as a layer of pervious electrospun nanofibers to generate more than 20 g/L concentrated H_2_O_2_. The process and electrode materials can be used in various H_2_O_2_-driven environmental remediation technologies.

## Supporting Information Available

Comparison of ORR-HP performance of composite GDE cathodes using different types and loadings of binders and catalysts, and operated at different current densities (PDF)
