# Supplementary material for: Electrosynthesis of >20 g/L H2O2 from Air
Source: ACS ES T Eng. 2021 Dec 14;2(2):242–50. doi: 10.1021/acsestengg.1c00366 (PMC8845047; doi:10.1021/acsestengg.1c00366)
Supplement: Supplementary file 1 — ee1c00366_si_001.pdf [file ee1c00366_si_001.pdf]

Supporting Information

Electrosynthesis of > 20 g/L H_2O_2 from air

Huihui Li,[1,2#] Estefanny Quispe-Cardenas,[2#] Shasha Yang,[2] Lifeng Yin,[1*] Yang Yang[2*]

[1] State Key Laboratory of Water Environment Simulation, School of Environment, Beijing Normal University, Beijing 100875, China

[2] Department of Civil and Environmental Engineering, Clarkson University, Potsdam, New York 13699, United States

[#] These authors contributed equally.

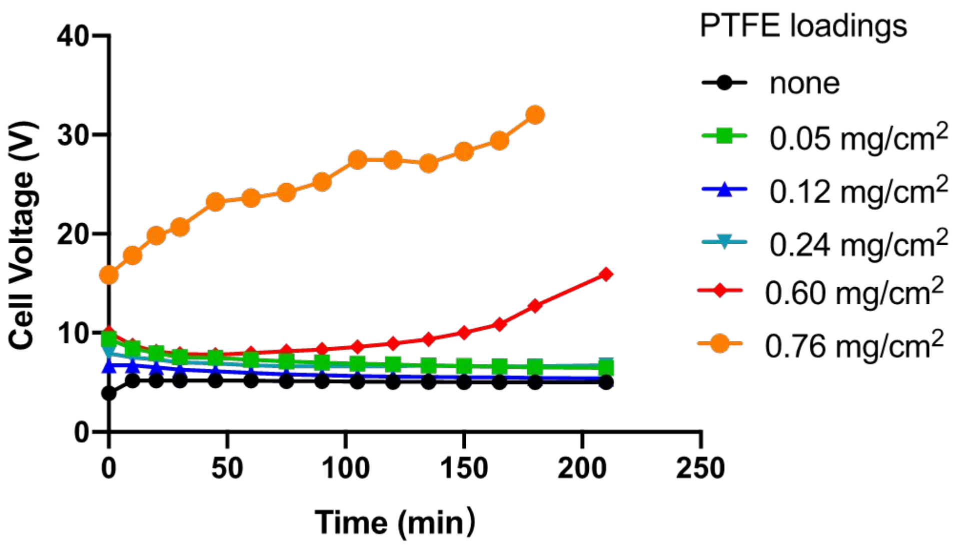

Figure S1. Impacts of PTFE (spray-coated) loadings on the cell voltages. The divided cell was operated at 12 mA/cm^2. Anolyte and catholyte are 0.1 M H_2SO_4 and 0.1 M $NaClO_4$, respectively.

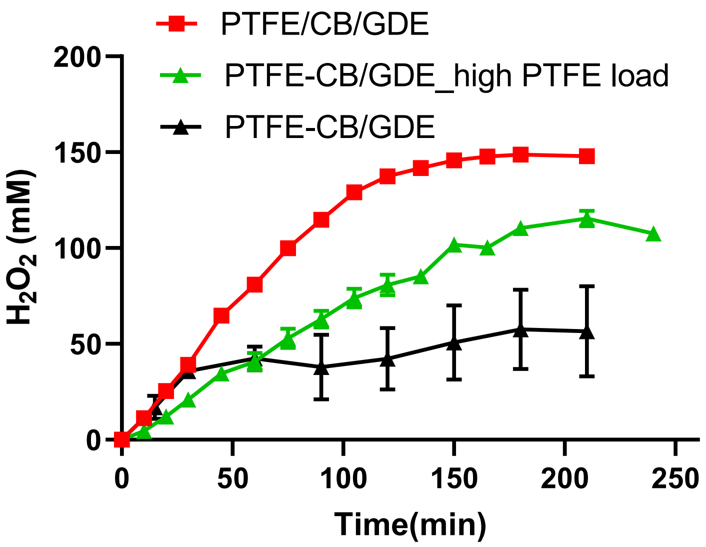

Figure S2. Impact of PTFE location (overcoat for PTFE/CB/GDE vs. blent-in for PTFE-CB/GDE) on H_2O_2 production. Tests were performed in the divided cell at 12 mA/cm^2. Anolyte and catholyte are 0.1 M H_2SO_4 and 0.1 M $NaClO_4$, respectively.

For all the samples, CB loading is controlled at 0.5 mg/cm^2. The PTFE loadings of PTFE/CB/GDE and PTFE-CB/GDE are 0.12 and 0.15 mg/cm^2, respectively. Notably, the PTFE-CB/GDE was prepared following the protocol reported previously with a slight modification:[1] The [PTFE]/[CB] mas ratio was kept the same as 1:3, only the CB loading was mandated as 0.5 mg/cm^2 to be consistent with other GDEs prepared in this study. However, we found that PTFE-CB/GDE had a stability issue. Triplicate tests exhibited significantly different performance in each run, resulting in large error bars (standard deviation) compared with others. To cope with this issue, the PTFE loading was increased to 4 mg/cm^2 to prepare sample "PTFE-CB/CB/GDE_High PTFE load".

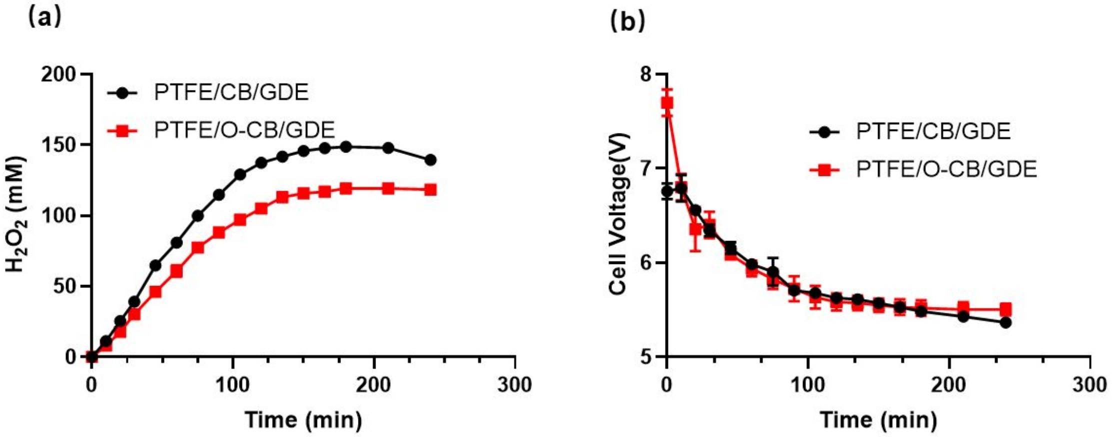

Figure S3. (a) ORR-HP performance and (b) cell voltage variation of cathodes using pristine or oxygenated CB (O-CB). The divided cell was operated at 12 mA/cm^2. Anolyte and catholyte are 0.1 M H_2SO_4 and 0.1 M $NaClO_4$, respectively.

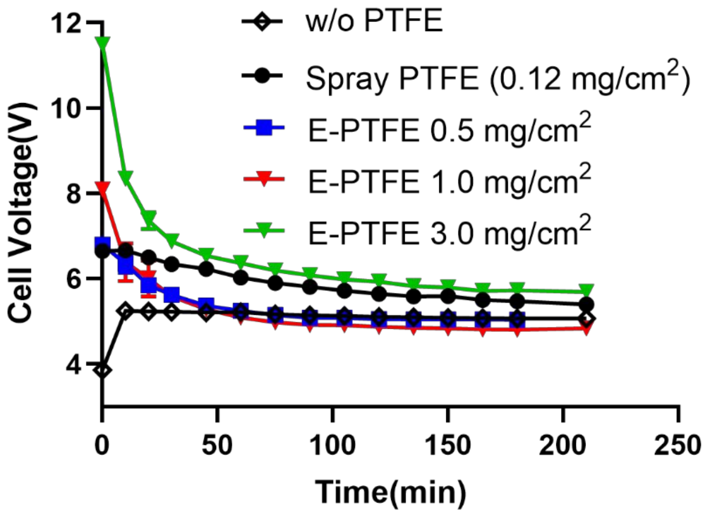

Figure S4. Variation of cell voltages during ORR-HP processes using cathodes with different types and loadings of PTFE coatings. All tests were performed at a current density of 12 mA/cm^2.

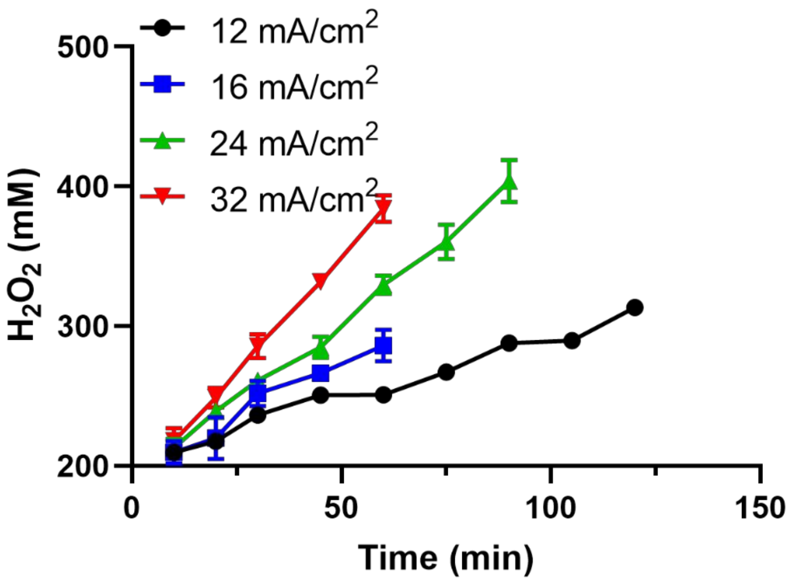

Figure S5. Impact of applied current density on the ORR-HP performance of E-PTFE/CB/GDE cathode. 200 mM H_2O_2 was spiked to the catholyte (100 mM $NaClO_4$) before electrolysis. If the cathodic H_2O_2 decomposition prevailed the H_2O_2 production, the decrease of [H_2O_2] should be observed. The net increase of [H_2O_2] at various current densities indicates that E-PTFE/CB/GDE has good performance on the production and preservation of H_2O_2.

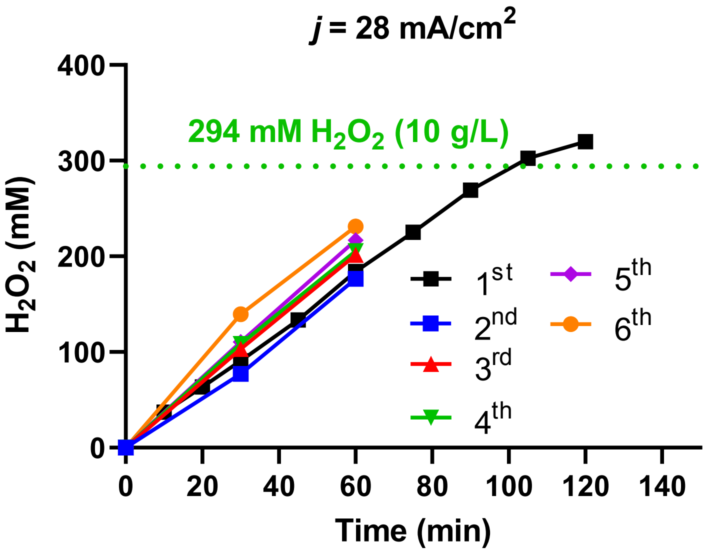

Figure S6. Repetitive testing of E-PTFE/CB/GDE. for the production of H_2O_2.

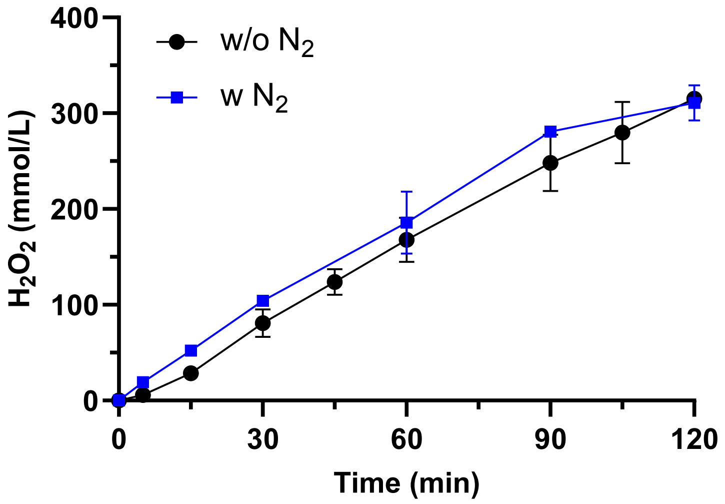

Figure S7. Comparison of ORR-HP performance in the presence and absence of N_2 purging. Test “w/o N_2” was operated in a divided cell equipped with E-PTFE/CB/GDE at 28 mA/cm^2. As for test “w/ N_2”, a stream of N_2 (*ca.* 100 mL/min) was purged into the catholyte to repel dissolved oxygen, while other operations remained the same.

Table S1. Literature data of H_2O_2 production performance using GDEs.

Cathode and Area	Catalyst	Current or Potential	Atmosphere; flowrate/ pressure	Production rates *		Current Efficiency	Max [H_2O_2]; Time to reach	Ref.
				mM cm^{-2} h^{-1}	mmol cm^{-2} h^{-1}			
GDE, 12.56 cm^2	Carbon black(CB)	28 mA/cm^2	Air; diffusion	15.0	0.45	86.8%	10,800 mg/L; 2 h	This work
GDE, 12.56 cm^2	Carbon black(CB)	28 mA/cm^2	Air; diffusion	19.0	0.57	86.6%	25,000 mg/L; 3.5 h	This work
GDE, 7.1 cm^2	CB	7.1 mA/ cm^2	Air; 0.5 L/min	0.8	0.16	59.4%	556 mg/L; 3 h	[2]
GDE, 4 cm^2	Acetylene black	20 mA/cm^2	Air; 2 L/min	1.73	0.34	92.7%	353 mg/L; 1.5 h	[3]
GDE, 6.15 cm^2	—	30 mA/cm^2	Air; 1.2 L/min	1.5	0.38	53%	330 mg/L; 1 h	[4]
GDE, 10 cm^2	CB	60 mA/cm^2	Air; 0.04 L/min	13.7	0.27	65%	9,367 mg/L; 2 h	[5]
GDE, 20 cm^2	FePc-CB	-1.0 V (vs./AgCl)	Air; diffusion	0.5	0.2	78.2%	240 mg/L; 1.5 h	[6]
GDE, 7.1 cm^2	TBAQ-CNTs	7.1 mA/cm^2	Air; 0.4 L/min	0.4	0.12	95%	151 mg/L; 1.5 h	[7]
GDE, 1 cm^2	Acetylene black	108 mA/cm^2	Air; 2 L/min	6.38	1.60	79.1%	678 mg/L; 2.5 h	[8]
GDE, 8 cm^2	Nitrogen-doped carbon	60 mA/cm^2	Air; 36 L/h	0.32	0.05	36.0%	520 mg/L; 6 h	[9]
GDE, 25 cm^2	CB	1 mA/cm^2	Air; diffusion	0.41	0.02	27.3%	1,035 mg/L; 3 h	[10]
GDE, 7 cm^2	CNT and NCB	30 mA/cm^2	Air; 1.5 L/min for 10 min	1.09	0.22	50.2%	520 mg/L; 2 h	[11]
GDE, 1.3 cm^2	Mesoporous carbon	150 mA/cm^2	Air; 1 psi	2.08	0.42	50%-55%	369 mg/L; 4 h	[12]
GDE, 14 cm^2	CB	35mA/cm^2	Air/O_2/N_2; 0.52 L/min	0.97	0.34	89%	461 mg/L; 1 h	[13]
GDE, 5.1 cm^2	CB	240 mA/cm^2	Air; diffusion	11.96	2.99	67%	3437 mg/L; 2h	[14]
GDE, 7.1 cm^2	Graphene	29 mA/cm2	Air; 0.2 L/min	0.68	0.27	43.5%	495 mg/L; 3 h	[15]
GDE, 4 cm^2	CB-10%	200 mA/cm^2	O_2; 50 ccm	3.4	0.09	>90%	200,000 mg/L	[16]
GDE, 49 cm^2	Co–C	8.2 mA/cm^2	O_2; 1000 mL/min	0.2	0.2	30.0%	1,400 mg/L; 72 h	[17]
GDE, 20 cm^2	CB	-1.1 V (vs./AgCl)	O_2; 0.2 bar	1.9	0.76	33.3%	6,424 mg/L; 5 h	[18]
GDE, 20 cm^2	—	-2.25 V (vs./AgCl)	O_2; 0.2 bar	0.3	0.06	—	414 mg/L; 2 h	[19]
GDE, 20 cm^2	TBAQ-CB	-1.0 V (vs. SCE)	O_2; 0.20 bar	0.4	0.16	89.60%	301 mg/L; 1 h	[20]
GDE, 12.75 cm^2	Acetylene black	20 mA/cm^2	O_2; 1-5 Mpa	0.93-1.66	0.28-0.50	~32%	719 mg/L; 1 h	[21]
GDE, 2 cm^2	—	10 mA/cm^2	Saturated O_2 solution	0.35	0.04	23.94%	48 mg/L; 1 h	[22]

GDE, 20 cm^2	Carbon (modified)	75 mA/cm^2	O_2; 0.2 bar	0.47	0.21	92.90%	555 mg/L; 2 h	[23]
GDE, 70.5 cm^2	Sudan Red 7B	75 mA/cm^2	O_2; 0.3 bar	0.93	0.23	17.87%	1,020 mg/L; 1.5 h	[24]
GDE, 3.0 cm^2	CoS_2/MWCNTs	100 mA/cm^2	O_2; 0.5 L/min	6.3	1.58	82%	1,935 mg/L; 3 h	[25]

* The production rate was defined as the average cumulative concentration of H_2O_2 per cathode area in 1 hour.

Text S1

For 35 wt% H_2O_2, its molar concentration can be calculated by the following equation:

$$n = \frac{1000\rho\omega}{M}$$

Where ρ (1.13 g/cm^3) is the density of 35 wt.% H_2O_2; M (34 g/mol) is the molar mass of H_2O_2. Thus, the molar concentration (n) of 35 wt.% H_2O_2 is 11.6 mol/L.

Assuming a 100% current efficiency in the ORR-HP reaction. The reaction time (*t*) to produce 11.6 mol/L H_2O_2 can be calculated as follows:

$$t = n'FcV/I$$

Where current *I* (A) equals 10 A/cm^2 × 10 cm^2 = 100 A; n' = 2 is the number of electrons in ORR-HP reaction. F (96485 C/mol) is the Faraday constant; c (11.6 mol/L) is the concentration of 35 wt.% H_2O_2; V (0.03 L) is the volume of the electrolyte.

The t is given as 671 s ≈ 11 min.

Text S2

The specific energy consumption (EC) for H_2O_2 production in 100 mM $NaClO_4$ is calculated based on the performance of E-PTFE (0.1 mg/cm^2)/CB/GDE shown in **Figure 5a** using the equation below:

$$EC = \frac{UIt}{cV}$$

Where U (4.8 V) is the cell voltage; I (0.15 A) is the applied current; t (4.5 h) is the electrolysis time; c (216 mM = 7.34 g/L) is the H_2O_2 concentration; V (0.03 L) is the electrolyte volume. Therefore, the EC is given as 14.8 kWh/kg.

Similarly, the ORR-HP in 30 mL lake water is operated at 15 V. 12.6 A/L specific charge is required to obtain 175 mM H_2O_2 (**Figure 7**). Based on the above parameters, the EC is calculated as 32 kWh/kg.

References

(1) Barazesh, J. M.; Hennebel, T.; Jasper, J. T.; Sedlak, D. L. Modular Advanced Oxidation Process Enabled by Cathodic Hydrogen Peroxide Production. *Environ. Sci. Technol.* **2015**, *49* (12), 7391–7399. https://doi.org/10.1021/acs.est.5b01254.

(2) Yu, X.; Zhou, M.; Ren, G.; Ma, L. A Novel Dual Gas Diffusion Electrodes System for Efficient Hydrogen Peroxide Generation Used in Electro-Fenton. *Chem. Eng. J.* **2015**, *263*, 92–100. https://doi.org/10.1016/j.cej.2014.11.053.

(3) Sheng, Y.; Song, S.; Wang, X.; Song, L.; Wang, C.; Sun, H.; Niu, X. Electrogeneration of Hydrogen Peroxide on a Novel Highly Effective Acetylene Black-PTFE Cathode with PTFE Film. *Electrochimica Acta* **2011**, *56* (24), 8651–8656. https://doi.org/10.1016/j.electacta.2011.07.069.

(4) Panizza, M.; Cerisola, G. Electrochemical Generation of H2O2 in Low Ionic Strength Media on Gas Diffusion Cathode Fed with Air. *Electrochimica Acta* **2008**, *54* (2), 876–878. https://doi.org/10.1016/j.electacta.2008.07.063.

(5) Luo, H.; Li, C.; Wu, C.; Zheng, W.; Dong, X. Electrochemical Degradation of Phenol by in Situ Electro-Generated and Electro-Activated Hydrogen Peroxide Using an Improved Gas Diffusion Cathode. *Electrochimica Acta* **2015**, *186*, 486–493. https://doi.org/10.1016/j.electacta.2015.10.194.

(6) Silva, F. L.; Reis, R. M.; Barros, W. R. P.; Rocha, R. S.; Lanza, M. R. V. Electrogeneration of Hydrogen Peroxide in Gas Diffusion Electrodes: Application of Iron (II) Phthalocyanine as a Modifier of Carbon Black. *J. Electroanal. Chem.* **2014**, *722–723*, 32–37. https://doi.org/10.1016/j.jelechem.2014.03.007.

(7) Lu, X.; Zhou, M.; Li, Y.; Su, P.; Cai, J.; Pan, Y. Improving the Yield of Hydrogen Peroxide on Gas Diffusion Electrode Modified with Tert-Butyl-Anthraquinone on Different Carbon Support. *Electrochimica Acta* **2019**, *320*, 134552. https://doi.org/10.1016/j.electacta.2019.07.063.

(8) Sheng, Y.; Zhao, Y.; Wang, X.; Wang, R.; Tang, T. Electrogeneration of H2O2 on a Composite Acetylene Black–PTFE Cathode Consisting of a Sheet Active Core and a Dampproof Coating. *Electrochimica Acta* **2014**, *133*, 414–421. https://doi.org/10.1016/j.electacta.2014.04.071.

(9) Daniel, G.; Zhang, Y.; Lanzalaco, S.; Brombin, F.; Kosmala, T.; Granozzi, G.; Wang, A.; Brillas, E.; Sirés, I.; Durante, C. Chitosan-Derived Nitrogen-Doped Carbon Electrocatalyst for a Sustainable Upgrade of Oxygen Reduction to Hydrogen Peroxide in UV-Assisted Electro-Fenton Water Treatment. *ACS Sustain. Chem. Eng.* **2020**, *8* (38), 14425–14440. https://doi.org/10.1021/acssuschemeng.0c04294.

(10) Murawski, E.; Kananizadeh, N.; Lindsay, S.; Rao, A. M.; Popat, S. C. Decreased Gas-Diffusion Electrode Porosity Due to Increased Electrocatalyst Loading Leads to Diffusional Limitations in Cathodic H2O2 Electrosynthesis. *J. Power Sources* **2021**, *481*, 228992. https://doi.org/10.1016/j.jpowsour.2020.228992.

(11) Lu, J.; Liu, X.; Chen, Q.; Zhou, J. Coupling Effect of Nitrogen-Doped Carbon Black and Carbon Nanotube in Assembly Gas Diffusion Electrode for H2O2 Electro-Generation and Recalcitrant Pollutant Degradation. *Sep. Purif. Technol.* **2021**, *265*, 118493. https://doi.org/10.1016/j.seppur.2021.118493.

(12) Garza-Campos, B.; Morales-Acosta, D.; Hernández-Ramírez, A.; Guzmán-Mar, J. L.; Hinojosa-Reyes, L.; Manríquez, J.; Ruiz-Ruiz, E. J. Air Diffusion Electrodes Based on Synthetized Mesoporous Carbon for Application in Amoxicillin Degradation by Electro-Fenton and Solar Photo Electro-Fenton. *Electrochimica Acta* **2018**, *269*, 232–240. https://doi.org/10.1016/j.electacta.2018.02.139.

(13) An, J.; Li, N.; Zhao, Q.; Qiao, Y.; Wang, S.; Liao, C.; Zhou, L.; Li, T.; Wang, X.; Feng, Y. Highly Efficient Electro-Generation of H2O2 by Adjusting Liquid-Gas-Solid Three Phase

Interfaces of Porous Carbonaceous Cathode during Oxygen Reduction Reaction. *Water Res.* **2019**, *164*, 114933. https://doi.org/10.1016/j.watres.2019.114933.

(14) Zhang, Q.; Zhou, M.; Ren, G.; Li, Y.; Li, Y.; Du, X. Highly Efficient Electrosynthesis of Hydrogen Peroxide on a Superhydrophobic Three-Phase Interface by Natural Air Diffusion. *Nat. Commun.* **2020**, *11* (1), 1–11. https://doi.org/10.1038/s41467-020-15597-y.

(15) Garcia-Rodriguez, O.; Lee, Y. Y.; Olvera-Vargas, H.; Deng, F.; Wang, Z.; Lefebvre, O. Mineralization of Electronic Wastewater by Electro-Fenton with an Enhanced Graphene-Based Gas Diffusion Cathode. *Electrochimica Acta* **2018**, *276*, 12–20. https://doi.org/10.1016/j.electacta.2018.04.076.

(16) Xia, C.; Xia, Y.; Zhu, P.; Fan, L.; Wang, H. Direct Electrosynthesis of Pure Aqueous H2O2 Solutions up to 20% by Weight Using a Solid Electrolyte. *Science* **2019**, *366* (6462), 226–231. https://doi.org/10.1126/science.aay1844.

(17) Li, W.; Bonakdarpour, A.; Gyenge, E.; Wilkinson, D. P. Drinking Water Purification by Electrosynthesis of Hydrogen Peroxide in a Power-Producing PEM Fuel Cell. *ChemSusChem* **2013**, *6* (11), 2137–2143. https://doi.org/10.1002/cssc.201300225.

(18) Barros, W. R. P.; Ereno, T.; Tavares, A. C.; Lanza, M. R. V. In Situ Electrochemical Generation of Hydrogen Peroxide in Alkaline Aqueous Solution by Using an Unmodified Gas Diffusion Electrode. *ChemElectroChem* **2015**, *2* (5), 714–719. https://doi.org/10.1002/celc.201402426.

(19) Reis, R. M.; Beati, A. A. G. F.; Rocha, R. S.; Assumpção, M. H. M. T.; Santos, M. C.; Bertazzoli, R.; Lanza, M. R. V. Use of Gas Diffusion Electrode for the In Situ Generation of Hydrogen Peroxide in an Electrochemical Flow-By Reactor. *Ind. Eng. Chem. Res.* **2012**, *51* (2), 649–654. https://doi.org/10.1021/ie201317u.

(20) Valim, R. B.; Reis, R. M.; Castro, P. S.; Lima, A. S.; Rocha, R. S.; Bertotti, M.; Lanza, M. R. V. Electrogeneration of Hydrogen Peroxide in Gas Diffusion Electrodes Modified with Tert-Butyl-Anthraquinone on Carbon Black Support. *Carbon* **2013**, *61*, 236–244. https://doi.org/10.1016/j.carbon.2013.04.100.

(21) Tan, X.; Jin, C.; Sun, W.; Zhao, Y.; Wei, H.; Sun, C. Synergetic Electrocatalytic Degradation of Isophorone by Active Oxygen Species Generated in the Gas Diffusion Electrode and PbO2 Anode. *Chemosphere* **2021**, *275*, 130060. https://doi.org/10.1016/j.chemosphere.2021.130060.

(22) Deng, F.; Li, S.; Cao, Y.; Fang, M. A.; Qu, J.; Chen, Z.; Qiu, S. A Dual-Cathode Pulsed Current Electro-Fenton System: Improvement for H2O2 Accumulation and Fe3+ Reduction. *J. Power Sources* **2020**, *466*, 228342. https://doi.org/10.1016/j.jpowsour.2020.228342.

(23) Kronka, M. S.; Silva, F. L.; Martins, A. S.; Almeida, M. O.; Honório, K. M.; Lanza, M. R. V. Tailoring the ORR Selectivity for H_2O_2 Electrogeneration by Modification of Printex L6 Carbon with 1,4-Naphthoquinone: A Theoretical, Experimental and Environmental Application Study. *Mater. Adv.* **2020**, *1* (5), 1318–1329. https://doi.org/10.1039/D0MA00290A.

(24) Moreira, J.; Bocalon Lima, V.; Athie Goulart, L.; Lanza, M. R. V. Electrosynthesis of Hydrogen Peroxide Using Modified Gas Diffusion Electrodes (MGDE) for Environmental Applications: Quinones and Azo Compounds Employed as Redox Modifiers. *Appl. Catal. B Environ.* **2019**, *248*, 95–107. https://doi.org/10.1016/j.apcatb.2019.01.071.

(25) Ridruejo, C.; Alcaide, F.; Álvarez, G.; Brillas, E.; Sirés, I. On-Site H2O2 Electrogeneration at a CoS2-Based Air-Diffusion Cathode for the Electrochemical Degradation of Organic Pollutants. *J. Electroanal. Chem.* **2018**, *808*, 364–371. https://doi.org/10.1016/j.jelechem.2017.09.010.
